# Mechanistic insights into the more potent effect of KP-54 compared to KP-10 *in vivo*

**DOI:** 10.1371/journal.pone.0176821

**Published:** 2017-05-02

**Authors:** Xavier d'Anglemont de Tassigny, Channa Jayasena, Kevin G. Murphy, Waljit S. Dhillo, William H. Colledge

**Affiliations:** 1 Reproductive Physiology Group, Department of Physiology, Development and Neuroscience, University of Cambridge, Cambridge, United Kingdom; 2 Section of Endocrinology and Investigative Medicine, Division of Diabetes, Endocrinology and Metabolism, Imperial College London at Hammersmith Campus, Commonwealth Building, London, United Kingdom; John Hopkins University School of Medicine, UNITED STATES

## Abstract

Kisspeptins regulate the mammalian reproductive axis by stimulating release of gonadotrophin releasing hormone (GnRH). Different length kisspeptins (KP) are found of 54, 14, 13 or 10 amino-acids which share a common C-terminal 10-amino acid sequence. KP-54 and KP-10 have been widely used to stimulate the reproductive axis but data suggest that KP-54 and KP-10 are not equally effective at eliciting reproductive hormone secretion after peripheral delivery. To confirm this, we analysed the effect of systemic administration of KP-54 or KP-10 on luteinizing hormone (LH) secretion into the bloodstream of male mice. Plasma LH measurements 10 min or 2 hours after kisspeptin injection showed that KP-54 can sustain LH release far longer than KP-10, suggesting a differential mode of action of the two peptides. To investigate the mechanism for this, we evaluated the pharmacokinetics of the two peptides *in vivo* and their potential to cross the blood brain barrier (BBB). We found that KP-54 has a half-life of ~32 min in the bloodstream, while KP-10 has a half-life of ~4 min. To compensate for this difference in half-life, we repeated injections of KP-10 every 10 min over 1 hr but failed to reproduce the sustained rise in LH observed after a single KP-54 injection, suggesting that the failure of KP-10 to sustain LH release may not just be related to peptide clearance. We tested the ability of peripherally administered KP-54 and KP-10 to activate c-FOS in GnRH neurons behind the blood brain barrier (BBB) and found that only KP-54 could do this. These data are consistent with KP-54 being able to cross the BBB and suggest that KP10 may be less able to do so.

## Introduction

Kisspeptins are a family of neuropeptides encoded by the *Kiss1* gene that are critical for activation of the mammalian reproductive axis at puberty [1] and regulation of ovulation in sexually mature females [2]. In humans, they are generated from a 145-amino acid precursor to produce smaller amidated peptides of 54, 14, 13 or 10-amino acids that all share the common C-terminal decapeptide sequence. Kisspeptins act via the G-protein coupled receptor, KISS1R (also known as GPR54) to stimulate GnRH release and subsequent secretion of gonadotrophic hormones (LH and FSH) in all mammalian species examined [3–8]. Inactivating mutations in *KISS1R* or *KISS1* cause hypogonadotrophic hypogonadism in humans and mice [1, 9–11].

The potency of the different kisspeptins has been examined both *in vitro* and *in vivo*. In general, all forms of kisspeptin show similar receptor binding affinities and cell signalling properties *in vitro* [12]. KP-10 exerts a potent direct depolarizing action on GnRH neurons and increases their firing rate in acute brain slices [13]. Moreover, KP-54 and KP-10 induce robust LH release after direct delivery into the brain with similar effects [3]. In contrast, KP-54 and KP-10 have different potencies when they are delivered systemically. This is particularly relevant for the proposed clinical use of kisspeptins, as they will most likely be administered peripherally rather than directly into the brain. Peripheral delivery of KP-54 stimulated release of LH over a sustained period of 1 to 4 hours, while KP-10 induced less sustained LH secretion between 10 min to 1 h [5, 14–18]. The reason for this difference in the potency of KP-54 and KP-10 after systemic delivery is not known. It may be caused by differences in the half-life of each peptide in the blood stream or by differences in their ability to cross the blood-brain barrier. We have directly compared KP-54 and KP-10 to gain an insight into why KP-54 elicits more sustained responses than KP-10 after systemic delivery. This knowledge will be relevant when considering which form of kisspeptin to use in a clinical setting and in understanding the mechanisms of action of the different kisspeptins.

## Materials and methods

### Study 1: Effect of peripheral administration of KP-54 or KP-10 on plasma LH levels in male mice

Male mice received a single intraperitoneal (i.p.) injection of 100 μl of PBS (vehicle control; 137 mM NaCl, 2.7 mM KCl, 10 mM Na_2_HPO_4_, 1.8mM KH_2_PO_4_, pH 7.4), KP-10 (1 nmol), or KP-54 (1 nmol) in PBS and LH levels in the plasma measured at 10 min and 2 h after treatment to compare early and late responses points. We chose the 10 min point as some studies have shown that KP-10 injection results in maximal LH secretion after 10–15 min [16, 18]. 100 μl of blood was collected from a tail vein 10 min after injection, mixed with 5 μl EDTA and centrifuged at 10,000 x g for 10 min at 4°C. The plasma supernatant was stored at -20°C until assayed. Two hours after injection, mice were deeply anaesthetized with pentobarbital (100mg/kg i.p.), ~300 μl of blood was rapidly collected from the vena cava and processed as described above. Finally the mice were perfused intracardially with 20 ml of 4% paraformaldehyde in PBS. Brains were removed and incubated for 1 h in the same fixative as for perfusion and placed in PBS for sectioning and immunohistochemistry processing.

### Study 2: Effect of peripheral administration of a high dose or multiple injections of KP-10 on plasma LH levels in male mice

Male mice received a single intraperitoneal (i.p.) injection of 100 μl of PBS (vehicle control), KP-10 (1 nmol), KP-10 (10 nmol), KP-54 (1 nmol) or a series of six i.p. injections (every 10 minutes over one hour) of KP-10 (1 nmol) to extend the duration of action of KP-10. 100 μl of blood was collected from a tail vein 10 min after the final injection and the mice were killed 2 hours after treatment by an injection of pentobarbital (100mg/kg i.p.). Blood was collected from the vena cava, mixed with 5 μl EDTA, centrifuged at 10,000 x g for 10 min at 4°C to obtain the plasma fraction, and stored at -20°C until assayed.

### Study 3: Effect of peripheral administration of KP-54 or KP-10 on c-FOS neuronal activation in the hypothalamus

The aim of this study was:

1. To determine areas in the hypothalamus in which peripheral administration of KP-54 or KP-10 activates c-FOS in GnRH neurons
2. To determine if peripheral administration of KP-54 or KP-10 activates c-FOS in GnRH neurons in areas of the hypothalamus that are behind the blood brain barrier.

Induction of expression of the immediate early gene *c-Fos* is regarded as a marker of neuronal activation [19]. KISS1R activation results in c-FOS induction, which allows identification of the GnRH neurons that are activated by KP. We examined co-localization of c-FOS protein in GnRH neurons by immunohistochemistry 2h after peripheral injection of either KP-10 or KP-54. To define hypothalamic neurons behind the blood-brain-barrier (BBB), some mice were injected with bovine serum albumin conjugated with fluorescein isothiocyanate (BSA-FITC), a compound that can be used to detect microvascular leakage and does not cross the BBB [20]. 1 nmol KP-54 was injected first, followed by injection of 100 μl of a 50mg/ml solution of BSA-FITC into the tail vein. The mice were perfused with 4% paraformaldehyde/PBS 2 hrs after KP-54 injection and the brains processed for c-FOS, GnRH and BSA-FITC detection. A negative control for fluorescence was also performed with unconjugated BSA.

### Study 4: To determine the pharmacokinetics of plasma kisspeptin levels following peripheral KP-54 or KP-10 administration in male mice

Male mice received a single i.p. injection of 1 nmol KP-10 or 1 nmol KP-54. 100 μl of blood was collected from the lateral tail vein from groups of mice (n = 4) for each time point (0, 1 min, 2 min, 5 min, 10 min, 30 min, 60 min and 120 min). Terminal bleeds were collected from the vena cava. Blood was mixed with 5 μl EDTA, centrifuged at 10,000 x g for 10 min at 4°C to obtain the plasma fraction, and stored at -20°C until assayed. Kisspeptin concentrations were measured using an established in house competitive radioimmunoassay (RIA, [5]. Briefly, this RIA uses a sheep polyclonal antibody (GQ2), raised against human KP-54, which cross-reacts with human KP-54, KP-14 and KP-10.

### Animals

3–5 month old wild-type (129S6/SvEv) male mice were used in this study. The mice were housed in open cages on a 12 hour light cycle (lights on at 0700 h) at a controlled temperature (22°C), and were provided with food and water *ad libitum*. All experiments were performed under the authority of a United Kingdom Home Office Project License and were approved by a Cambridge Animal Ethics Committee.

### Reagents

KP-10 [human Metastin (45–54) amide, M-2816] was purchased from Sigma-Aldrich. KP-10 consists of the C-terminal 10-amino acids of KP-54. Human KP-54 was synthesized by the Advanced Biotechnology Centre, Imperial College, London, UK. BSA-FITC was purchased from Sigma (A9771). From the product data sheets, the solubility of KP-10 and KP-54 in water is 1mg/ml. We prepared stock solutions in sterile PBS at 100 μM, which is almost ten times lower than the solubility limit set by the manufacturer. The 100 μM stock solution was diluted in sterile PBS and 100 μl of the working solution was injected to each mouse. PBS was used for vehicle control injections.

The rabbit anti-GnRH antibody was a gift from Dr. Vincent Prévot (Institut National de la Santé et de la Recherche Médicale U837, University of Lille 2, Lille, France). The rabbit polyclonal anti c-FOS antibody (SC-52) was purchased from Santa Cruz Biotechnology and previously used on mouse tissue [21]. Secondary goat anti-rabbit biotin-conjugated (BA-1000), anti-rabbit peroxidase-conjugated (PI-1000), and Vectastain ABC kit (PK-4000) were purchased from Vector Laboratories.

### Immunohistochemistry

Free-floating, dual-label chromogen immunohistochemistry was undertaken as described previously [2, 21]. Mice were perfused with 4% paraformaldehyde in PBS and the brains removed and post-fixed for 1h in the same fixative. The brains were cut as 40 μm free-floating coronal sections using a vibratome (VT1000S; Leica). Sections containing GnRH neurons were taken from the region of the medial septum through to the caudal hypothalamus. Sections were treated with 3% hydrogen peroxide for 10 min to quench endogenous peroxidase activity and then washed in Tris buffered saline (TBS) (0.5 M Tris, pH 7.6 and 0.15 M sodium chloride). For the first immunolabelling, sections were incubated for 16 h at 4°C with a primary rabbit polyclonal anti-sera directed against c-FOS (sc-52, Santa Cruz Biotechnology, USA) at 1:1000 in TBS containing 0.3% Triton X-100 (TBS-T) and 5% normal goat serum (blocking solution). Sections were then incubated with biotinylated anti-rabbit IgG (Vector Laboratories, USA) at 1:500 in blocking solution for 1 h at room temperature. After subsequent washing in TBS, the sections were incubated in Vectastain ABC avidin–peroxidase (Vector Laboratories, USA) at 1:100 in TBS-T for 1 h at room temperature. Immunoreactivity was revealed using glucose–oxidase, nickel-enhanced diaminobenzidine hydrochloride (DAB) that resulted in a black precipitate within the nucleus of the labelled cell. For the second immunolabelling, sections were washed in 3% hydrogen peroxide to quench any remaining peroxidase, washed in TBS, and then incubated with polyclonal rabbit anti-GnRH antibody (1:3000) in blocking solution for 16 h at 4°C. The rabbit anti-GnRH antibody was a gift from Dr. Vincent Prévot (University of Lille, France) initially generated and validated by Professor Gerard Tramu [22]. Sections were then incubated in peroxidase-conjugated anti-rabbit IgG (Vector Laboratories, USA) (1:500) in blocking solution for 1 h at room temperature. Immunoreactivity was revealed using glucose oxidase and DAB without nickel to generate a brown precipitate within the cytoplasm. Brain sections were mounted on slides, dehydrated, and cover slipped with DPX (44581; Sigma-Aldrich).

### Immunohistochemistry analysis

Sections were examined using an Axioscope 2 plus microscope (Carl Zeiss) using bright-field or fluorescence microscopy. Analysis of the double-labelled tissue was undertaken by counting the number of single-labelled (brown cytoplasm only) and dual-labelled (brown cytoplasm and black nucleus) neurons. The examiner was blinded of the treatment at the time of counting. Where appropriate, BSA-FITC was detected by epifluorescence microscopy at an excitation wavelength of 490 nm and an emission wavelength of 525 nm (green). About fifteen successive sections containing GnRH neurons were counted per animal (n = 6 per treatment). GnRH neuron subpopulations were separated into four hypothalamic regions [23]: the medial septum plus the diagonal band nucleus (MS/NDB), the medial preoptic area and ventromedial preoptic nucleus (MPO/VMPO), the vascular organ of the lamina terminalis (OVLT), and the median preoptic nucleus (MEPO) according to the Allen Mouse Brain Atlas.

### Luteinizing hormone ELISA

Plasma LH was assayed using a commercial ELISA kit (LH Ultrasensitive Rodent ELISA, Endocrine Technologies Cat Number ERK 7010+) with a sensitivity of 0.3 ng/ml and 7% intra-assay and 10% inter-assay coefficients of variation. 50 μl plasma samples from the vena cava were assayed in duplicate, but samples from tail vein bleeds were assayed with one replicate owing to limited sample volume.

### Statistics

Differences between groups were analyzed by one-way ANOVA assuming a Gaussian distribution, followed with Tukey multiple comparison test for unequal replication. The comparison between two groups was analysed by an unpaired, two-tailed, Student’s t-test. A P-value of less than 0.05 was taken as significant.

## Results

### Peripheral administration of KP-54 or KP-10 has different effects on LH secretion in male mice

The aim of these experiments was to compare the effect of KP-54 or KP-10 on LH secretion *in vivo*.

#### Study 1

**Fig 1:**
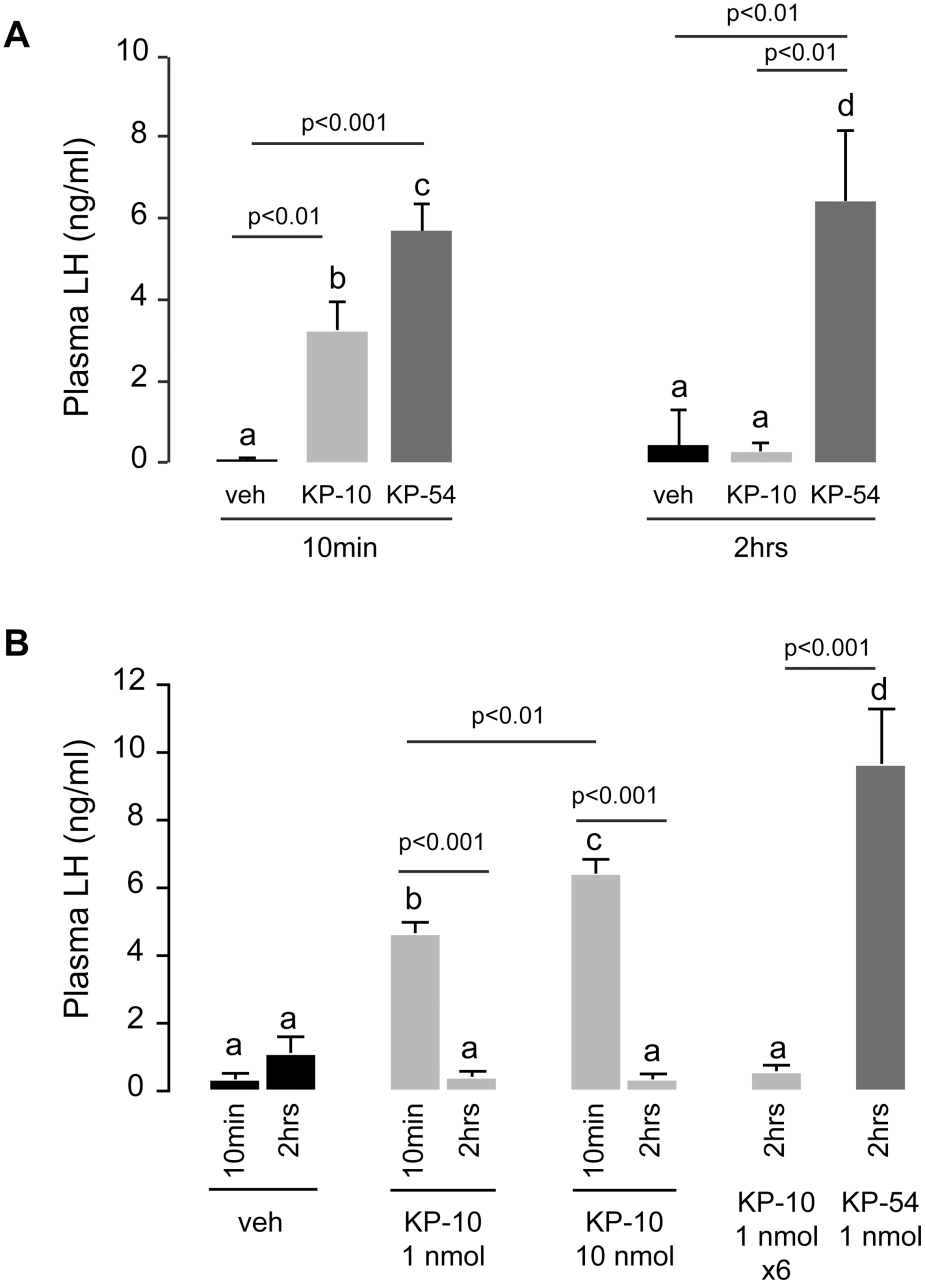
Peripheral administration of KP-54 stimulates plasma LH levels in male mice more potently than KP-10. (A) Bar graph showing the mean ± s.e.m. of LH levels in adult male mice 10 min and 2 hrs after intraperitoneal injection of PBS (veh, n = 8), 1 nmol KP-10 (n = 8), or 1 nmol KP-54 (n = 8). (B) Bar graph showing the mean ± s.e.m. of plasma LH levels 10 min and 2 hrs after intraperitoneal injection of PBS (n = 4), 1 nmol KP-10 (n = 5), 10 nmol (n = 4), 6 repeats of 1 nmol KP-10 every 10 min for 1h (n = 4), or 1 nmol KP-54 (n = 10). Letters indicate differences between groups at the p<0.05 significance level with higher P-values indicated (One-way ANOVA, followed by Tukey’s multiple comparison test was used to compare treatment groups).

In the first experiment, we assessed LH secretion after of a single intraperitoneal injection of KP-54 or KP-10 at the commonly used dose of 1 nmol (Fig 1A). In wild-type male mice, KP-54 increased plasma LH levels above vehicle control at 10 min (5.70 ± 0.69 ng/ml, *p* < 0.001) and at 2 hours (6.42 ± 1.74 ng/ml, *p* < 0.01) post-injection. KP-10 significantly increased LH levels at 10 min (3.22 ± 0.86 ng/ml, *p* < 0.01 vs. PBS treatment 0.06 ± 0.04 ng/ml), but to a significantly lower level than KP-54 at the same time point (*p* = 0.05). Two hours after KP-10 injection however, LH had returned to basal levels (0.25 ± 0.11 ng/ml, *p* < 0.01 vs KP-54 at 2 hrs and *p* < 0.01 vs KP-10 at 10 min).

#### Study 2

We performed a second series of experiments to test the effects of a 10-fold higher dose (10 nmol) or multiple injections of KP-10 (1nmol) on LH secretion (Fig 1B). A single injection of 10 nmol KP-10 resulted in a significantly greater increase in LH after 10 min (6.39 ± 0.37 ng/ml) than a single injection of 1 nmol KP-10 (4.62 ± 0.29 ng/ml, *p* < 0.01), but LH had returned to basal levels (0.31 ± 0.12 ng/ml) by 2 hrs. Similarly, six-repeated injections (every 10 minutes for 1h) of 1 nmol KP-10 failed to sustain LH at the 2 h time point (0.54 ± 0.14, ng/ml). In the same experiment, a single i.p. injection of 1 nmol KP-54 induced a robust and sustained LH release at 2 hrs (9.62 ± 1.67 ng/ml). We attempted to facilitate greater permeability of KP-10 across the blood brain barrier (BBB) by adding a penetratin sequence [24] to the C-terminal end of the decapeptide but this did not further increase plasma LH levels (S1 Fig).

### Peripheral KP-54 (but not KP-10) induces c-FOS in GnRH neurons located behind the blood brain barrier

#### Study 3

**Fig 2:**
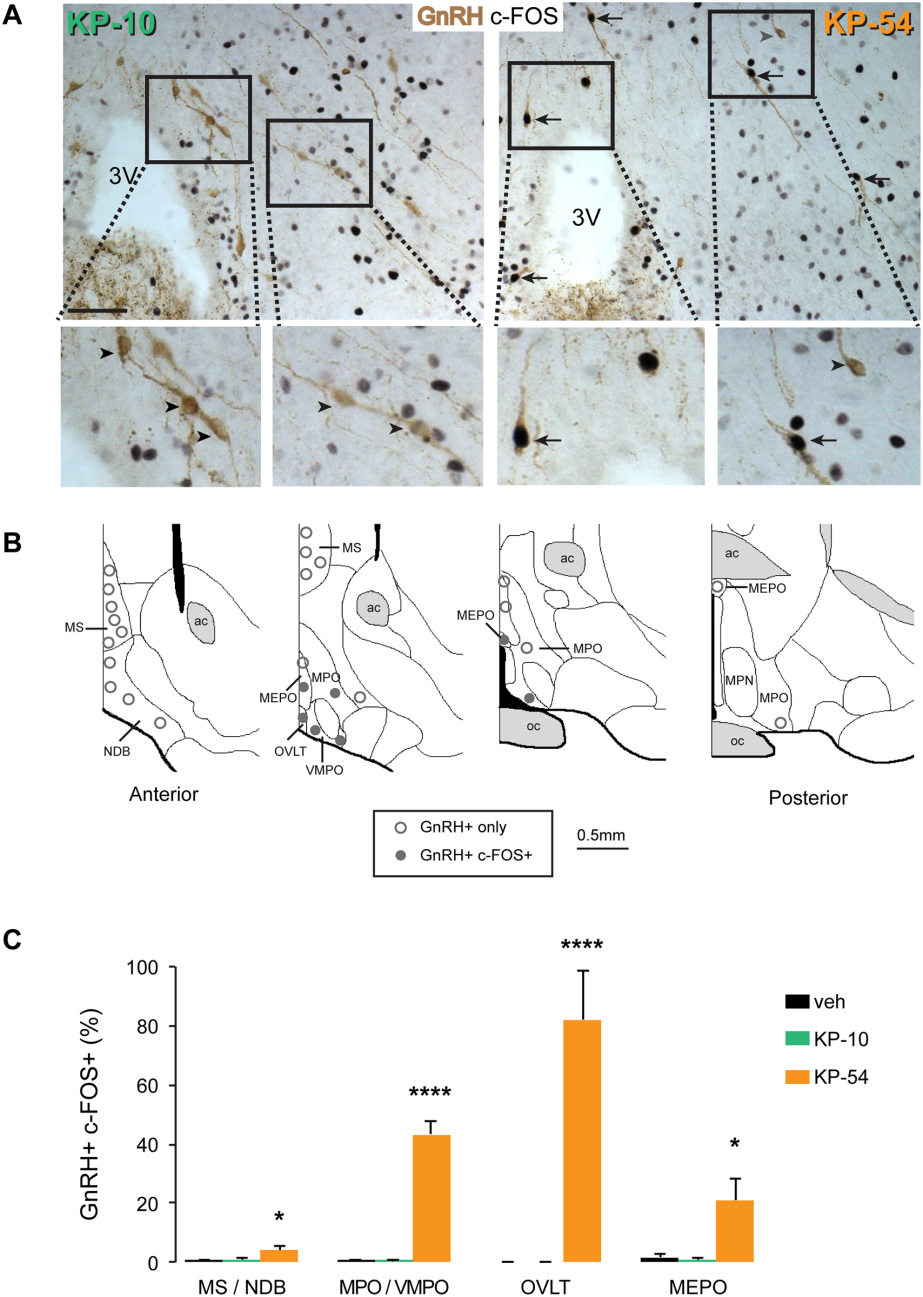
Peripheral administration of KP-54 but not KP-10 induces c-FOS in GnRH neurons. (A) Representative photomicrographs showing coronal sections of dual-labelled immunohistochemistry showing c-FOS staining (black nuclei) and GnRH neurons (brown cells) at the level of the OVLT and MPO regions in the mouse hypothalamus 2 hrs after peripheral injection of KP-54 (n = 6) or KP-10 (n = 6). GnRH neurons with c-FOS staining are indicated with black arrows. Grey arrowheads indicate c-FOS negative GnRH neurons. KP-54 (but not KP-10) stimulates c-FOS in the hypothalamus (and this co-localises with GnRH neurons). Scale bar = 20 μm. (B) Schematic diagrams showing distribution of c-FOS positive (closed circles) and c-FOS negative (open circles) GnRH neurons throughout the anterior hypothalamus following peripheral administration of KP-54 (n = 6). Diagrams are re-drawn from those available from the Allen Institute for Brain Science (www.alleninstitute.org/). ac anterior commissure; MEPO, median preoptic nucleus; MPN, medial preoptic nucleus; MPO, medial preoptic nucleus; MS, medial septum; NDB, diagonal band nucleus; oc, optic chiasm; OVLT, organum vasculosum of lamina terminalis; VMPO, ventromedial preoptic nucleus. (C) Histogram showing the number of GnRH neurons expressing c-FOS 2 hrs after PBS (veh, n = 6), KP-54 (n = 6) or KP-10 (n = 6) in different hypothalamic regions. MS/NDB, medial septum and diagonal band nucleus; MPO/VMPO, medial preoptic area and ventromedial preoptic nucleus; OVLT, organum vasculosum of lamina terminalis; MEPO, median preoptic nucleus. Significant differences between KP-54 and vehicle or KP-10 are indicated (one-way ANOVA, followed by Tukey’s multiple comparison test).

**Fig 3:**
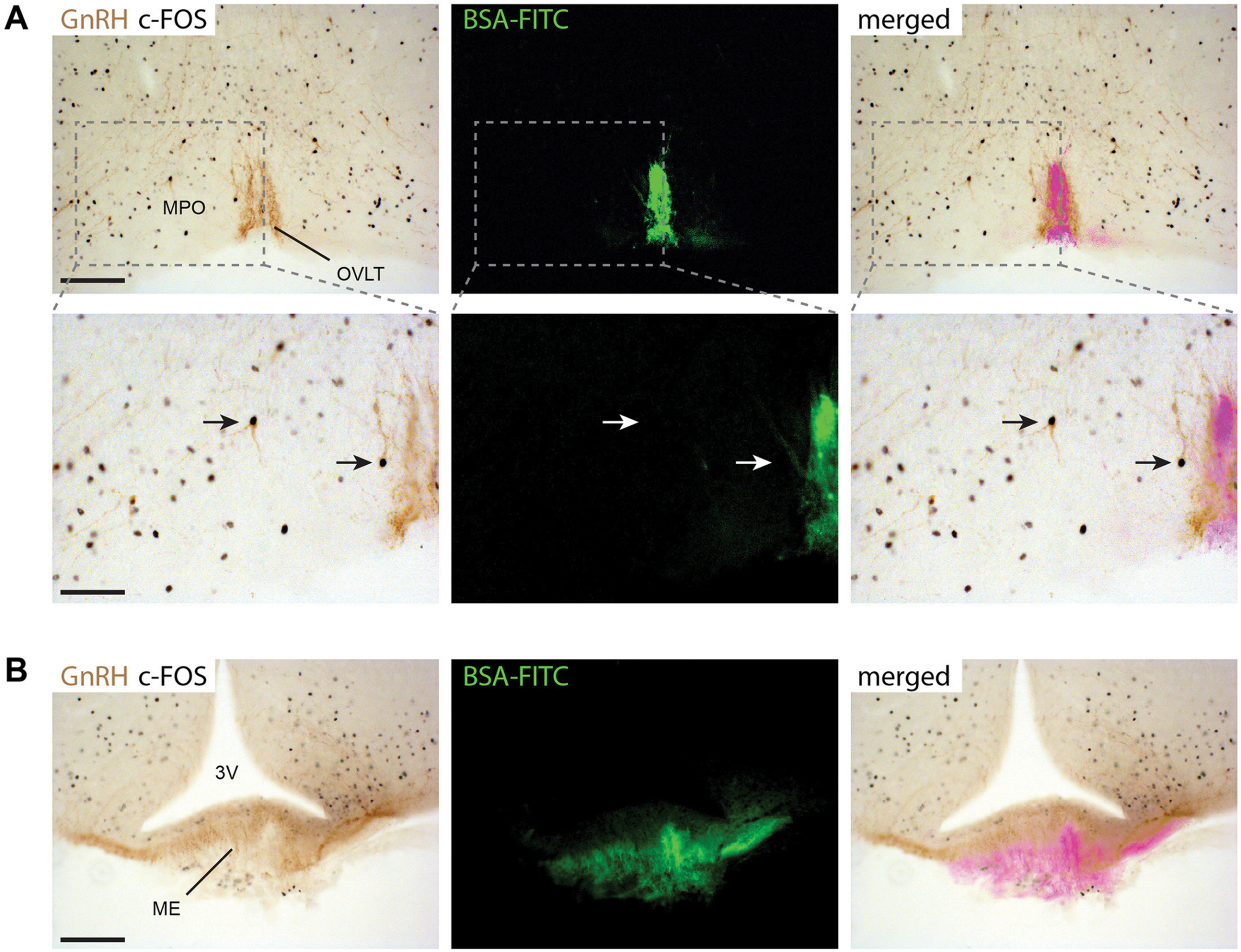
Peripheral administration of KP-54 activates GnRH neurons inside the blood-brain-barrier in male mice. KP-54 (1nmol) was injected into male mice (n = 3) and activation of c-FOS in GnRH neurons determined after 2 h (A) Dual-label immunohistochemistry showing GnRH neurons (brown cytoplasmic staining) and c-FOS staining (black nuclei) in the OVLT/MPO region. Low-power view (upper panels) and higher magnified views (lower panels) of the dotted line boxes show c-FOS + GnRH neurons (arrows) in the OVLT and MPO hypothalamic regions. The c-FOS + GnRH neuron in the MPO (arrow in the centre of the picture) is clearly located inside the blood brain barrier defined by BSA-FITC staining. Scale bars: 100 μm (upper panels) and 50 μm (lower panels). (B) GnRH axon projections (brown) overlap with the BSA-FITC staining in the median eminence (EM) as this region is devoid of a blood-brain-barrier. Scale bar = 100 μm. 3V: third ventricle, OVLT: organum vasculosum of lamina terminalis, MPO: medial preoptic area.

To further explore the mechanistic differences between KP-10 and KP-54 in the stimulation of LH release, we assessed the induction of c-FOS in GnRH neurons throughout the hypothalamic preoptic region (Fig 2A). The percentage of GnRH cells with c-FOS immunoreactivity was calculated in the four main regions of the hypothalamus that contain GnRH cells: the medial septum and diagonal band nucleus (MS/NDB), the medial preoptic area and ventromedial preoptic nucleus (MPO/VMPO), the organum vasculosum of lamina terminalis (OVLT), and the median preoptic nucleus (MEPO) (Fig 2B), We found that the percentage of GnRH neurons that were positive for c-FOS was low after peripheral KP-10 injection (0.66 ± 0.19%) and similar to the percentage activated in vehicle-treated animals (0.54 ± 0.15%) (Fig 2C). However, KP-54 induced expression of c-FOS in 22.07 ± 2.96 of the GnRH neurons (Fig 2C). The percentage of dual-labelled cells varied depending on the hypothalamic region: 4.00 ± 1.38 in the MS/NDB, 43.34 ± 4.22 in the MPO/VMPO, 82.05 ± 16.46 in the OVLT, and 21.07 ± 7.26 in the MEPO (Fig 2C). In KP-54-treated mice that also received an intravenous injection of BSA-FITC, the resulting fluorescence was restricted to the OVLT and the median eminence (ME) hypothalamic regions (Fig 3). We observed c-FOS-positive GnRH neurons in hypothalamic areas that were negative for BSA-FITC such as the MPO.

### Peripheral administration of KP-54 results in a higher peak level and longer duration of plasma kisspeptin levels compared to KP-10 in male mice

#### Study 4

**Fig 4:**
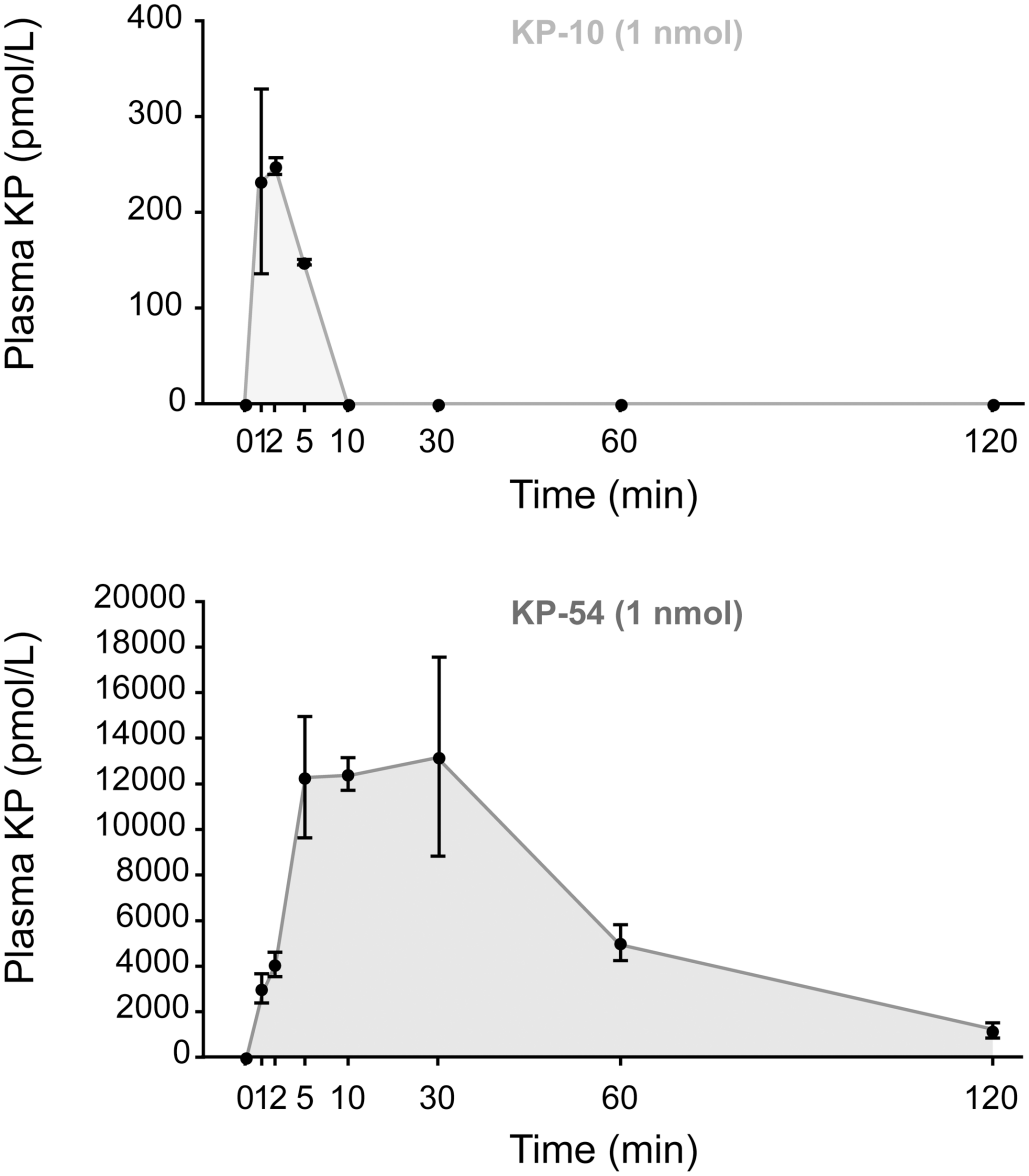
Peripheral administration of KP-54 results in a higher peak level and longer duration of plasma kisspeptin levels compared to KP-10 in male mice. Radioimmunoassay analysis of kisspeptin plasma levels over 2 hours after 1 nmole i.p. injection of KP-10 (n = 4) or KP-54 (n = 4). Note the different scale between the KP-10 and the KP-54 plots. Data points are means ± SEM.

To further explore the difference in the potencies of KP-10 and KP-54 on stimulating LH release, we measured the rate of loss of each peptide from the bloodstream following i.p. injection. KP-10 peaked at 248 ± 9 pmol/l at 2 min post-injection and was undetectable by 10 min (Fig 4). We estimate that KP-10 has a plasma half-life of about 4 min in the mouse. In contrast, plasma KP-54 increased rapidly after injection and reached a maximum at 5 min (12292 ± 2655 pmol/l) that lasted until 30 min (13193 ± 4364 pmol/L). Thereafter, plasma KP-54 level progressively diminished to 5034 ± 787 pmol/l at 60 min and 1186 ± 330 pmol/l at 120 min post-injection. We calculated the half-life of plasma KP-54 to be around 32 min in the mouse although as the RIA can recognise all forms of Kp, this may also reflect the immunoreactivity of shorter peptides produced by the breakdown of KP-54.

## Discussion

Kisspeptin neuropeptides are potent stimulators of GnRH release and are being evaluated for treatment of specific human reproductive disorders [25]. These clinical studies have used peripheral delivery of either the 54 amino-acid version of kisspeptin (KP-54)[5, 26–29] or the 10 amino-acid version (KP-10) [7, 8, 30]. Although KP-54 and KP-10 show similar receptor binding affinities and bioactivities *in vitro*, a direct comparison of their potencies *in vivo* is limited as most studies usually evaluate only one kisspeptin type. One study that compared KP-54 and KP-10 directly showed that injection into the brain produced similar levels of LH secretion in adult male mice after 30 mins [3]. Similarly in healthy men, continuous intravenous infusion of equimolar amounts of KP-10 or KP-54 over a 3h period also produced similar LH secretory responses [31]. Other data however, suggest that KP-54 and KP-10 have different potencies when they are delivered systemically as a single injection [15–17], which is the most likely route of administration in a clinical setting. In addition, whether KP-54 and KP-10 have different mechanisms of action after systemic delivery has not been studied.

In this study, we have examined the effect of a single injection of equimolar amounts of KP-54 or KP-10 on LH secretion and c-FOS activation of GnRH neurons in mice. We find that KP-10 is less potent than KP-54 at eliciting sustained LH release and our data suggest that this is caused by a combination of factors; the ability of KP-54, but not KP-10, to cross the blood brain barrier and a better pharmacokinetic profile for KP-54 compared to KP-10 in the bloodstream. It should be noted however that we used human KP-54 and KP-10 for these studies because the RIA does not detect mouse kisspeptins. We do not know if murine kisspeptins will behave differently.

Our data suggest that KP-10 may not cross the BBB. However, it is difficult to determine whether the lack of GnRH neuronal activation following peripheral administration reflects a lack of brain access, the low circulating half-life of KP-10 or a combination of both. One caveat to our study is that the method of determining the BBB permeability is based on the functional assay of c-FOS immunoreactivity rather than direct protein measurement. To determine the relative permeability of the BBB to KP-10 vs KP-54, it would be necessary to label each peptide and quantify entry into the brain. Direct measurement of kisspeptin entry into the brain, however, is not without its difficulties. For example, any label attached to the kisspeptin protein could alter its transport properties across the BBB. Radio-labelling of kisspeptins provides a sensitive detection method but radioisotopes in the brain could be derived from peptide degradation and not necessarily represent full-length kisspeptin. To overcome these technical challenges, we decided to use c-FOS activation in those GnRH neurons, which lie behind the BBB as a functional surrogate for entry of kisspeptins into the brain.

Our data show that the stability of KP-10 in the blood stream is short with a half-life of around 4 minutes, which is very similar to the 3.8 minutes found in men [5, 30]. In contrast, KP-54 had a half-life of 32 minutes in mice, which is close to the 28 minutes found in humans [5]. It should be noted, however that the RIA used to measure KP-54 may also detect shorter breakdown products of this protein that might be biologically inert. It is likely that the lack of GnRH neuronal activation and sustained LH release by KP-10 is mainly due to its instability. It is noteworthy that we failed to find c-FOS activation in GnRH neurons located close to the OVLT, which should be accessible to KP-10 from the bloodstream suggesting that KP-10 instability may be the biggest factor for its shorter potency *in vivo*. To try to overcome this limitation, we injected KP-10 every 10 minutes over a 1h period (6 injections in total), to extend the duration of the actions of KP-10. This did not extend the duration of LH secretion however, which supports the suggestion that KP-10 may not be able to cross the BBB, although more frequent or continuous delivery of KP-10 might be more efficacious. In contrast, KP-54, which produced sustained LH secretion over a 2h time period, activated GnRH neurons located behind the blood brain barrier in the medial preoptic area and median preoptic nucleus. This shows that KP-54 is capable of crossing the BBB and is consistent with other studies involving peripheral delivery of KP-54 which can induce c-FOS in GnRH neurons [14]. Indeed, the finding that intravenous KP-54 injection can enhance limbic brain activity in humans is also consistent with KP-54 crossing the BBB [32]. It should be noted that c-FOS activation in GnRH neurons located within 100 μm of the OVLT region cannot be used to define the BBB, since GnRH neurons in this location extend their dendrites outside the BBB [33].

Our data also show that intraperitoneal injections of kisspeptins can rapidly access the bloodstream with KP-10 achieving maximum levels after 2 minutes and KP-54 after 5 minutes (Fig 4). Interestingly, the peak level of KP-10 in the bloodstream (250 pmol/L) is almost 50-times lower than that of KP-54 (12000pmol/L) even though equimolar amounts (1 nmole) of each peptide were injected. Thus, it is possible that 250 pmol/L of KP-10 is below a critical threshold for c-FOS induction and it might be that a much higher dose is required.

If we assume that the total blood volume for a mouse is approximately 5ml, then only around 0.13% of KP-10 and 6% of KP-54 enter the bloodstream after intraperitoneal injection. It is possible that direct injection of kisspeptins into the bloodstream, which would achieve higher circulating levels might alter the responses we have observed. If we assume that KP-54 and KP-10 are equally competent to enter the bloodstream from the peritoneal space, then the 50-fold difference in peak levels between the two peptides might indicate that KP-10 is sequestered in some way, perhaps by rapid removal from the vascular space. It is also possible that the apparent short-half life of KP-10 may reflect sequestration by serum proteins, which would reduce the availability of the peptide for measurement by the RIA

The mechanism by which KP-54 might cross the BBB is not known. Substances cross the BBB by a variety of routes including via specific transporters and transmembrane diffusion. A BLAST protein search using the KP-54 amino-acid sequence did not identify any motifs that might bind to known BBB transporter proteins. The KP-54 protein also has no predicted glycosylation sites that might allow specific transport. The KP-54 protein also has good solubility in water making it unlikely that it will cross the BBB by transmembrane diffusion, which is usually restricted to hydrophobic proteins. Interestingly, KP-10 is very hydrophobic with 5/10 amino acids being aromatic or aliphatic, which would facilitate diffusion across the BBB, but this hydrophobicity might also cause sequestration by serum proteins and limit access to the brain.

The ability of KP-10 to cross the BBB is an important question as several KP-10 analogues have been developed to act as GPR54 agonists for manipulation of the reproductive axis and to treat clinical conditions including prostate cancer [34–42]. One of these KP-10 analogues (C6) has been shown to induce synchronized ovulations and allow pregnancies after a single intramuscular injection in sheep [36]. Interestingly, the LH responses with this KP-10-C6 analogue lasted up to 12 h, far longer than following systemic delivery of KP-54. It is likely that increased stability of the KP-10-C6 peptide accounts for this but whether the peptide can cross the BBB has not been studied. The C6 peptide contains an albumin-binding motif, which would reduce peptide clearance from the blood but may also limit access to the brain.

Our data indicate that peripheral injection of KP-10 can stimulate LH secretion but this is not sustained. We have shown previously using tissue explants that KP-10 can stimulate GnRH release from nerve terminals in the median eminence without the need for GnRH cell bodies [43]. In addition, immunoelectron microscopy has shown that kisspeptin nerve fibres make contact with GnRH nerve terminals in the median eminence [44] As we do not find c-FOS activation in GnRH cell bodies after KP-10 injection, this suggests that KP-10 may only be able to stimulate GnRH nerve terminals [35–42] leading to a rapid and transient stimulation of gonadotrophs in the anterior pituitary, thus resulting in a short stimulation of LH release. In contrast, systemic administration of KP-54 produces a robust and sustained LH secretion by acting directly at the GnRH neuron cell body, beyond the blood-brain-barrier.

Our data are relevant to the design of kisspeptin-based treatments for infertility problems such as hypothalamic amenorrhea [28, 29] or as safer alternatives for triggers of oocyte maturation for *in vitro* fertilization clinical protocols [27, 45]. KP-54 and KP-10 have been used clinically to stimulate the reproductive axis of men and women and they both show physiological effects. Our data suggest, however, that KP-54 might be more efficacious than Kp-10, particularly if using a regimen involving a single injection of peptide.

## Supporting information

S1 Fig Kisspeptin-penetratin treatment. This experiment aimed at facilitating Kp10 to cross the blood-brain-barrier by adding a penetratin extension [24] to the C-terminal end of Kp10, thereby putatively provided with higher permeability. Two penetratin variants were tested: the pen(ala2) residue has a lower protein transduction domain activity than pen-Kp10. The Kp10 peptide sequence used was Y N W N S F G L R F–NH2. The peptide sequences of modified Kp10 were: N R R M K W K K Y Y N W N S F G L R F–NH2 (Pen-Kp10), and N R R M A W A K Y Y N W N S F G L R F–NH2 (Pen(ala2)-Kp10). Each animal received a single 100 μl i.p. injection of either PBS, Kp10, Kp54, Pen-Kp10 or Pen(ala2)-Kp10. Blood samples were collected at 10 min (from tail vein) and 2 hrs (from vena cava upon sacrifice) time points after administration. The results from two animals per treatment (n = 2), suggest that adding a penetratin residue to the Kp10 decapeptide does not improve the Kp10-induced LH secretion.
(TIF)
