# Correction: Mechanistic insights into the more potent effect of KP-54 compared to KP-10 *in vivo*

**DOI:** 10.1371/journal.pone.0192014

**Published:** 2018-01-25

**Authors:** Xavier d’Anglemont de Tassigny, Channa N. Jayasena, Kevin G. Murphy, Waljit S. Dhillo, William H. Colledge

The second author’s name is incorrect. The correct name is: Channa N. Jayasena. The correct citation is: d’Anglemont de Tassigny X, Jayasena CN, Murphy KG, Dhillo WS, Colledge WH (2017) Mechanistic insights into the more potent effect of KP-54 compared to KP-10 *in vivo*. PLoS ONE 12(5): e0176821. https://doi.org/10.1371/journal.pone.0176821
