# Supplementary figures and images for: Mechanistic insights into the more potent effect of KP-54 compared to KP-10 in vivo
Source: PLoS One. 2017 May 2;12(5):e0176821. doi: 10.1371/journal.pone.0176821 (PMC5413024; doi:10.1371/journal.pone.0176821)

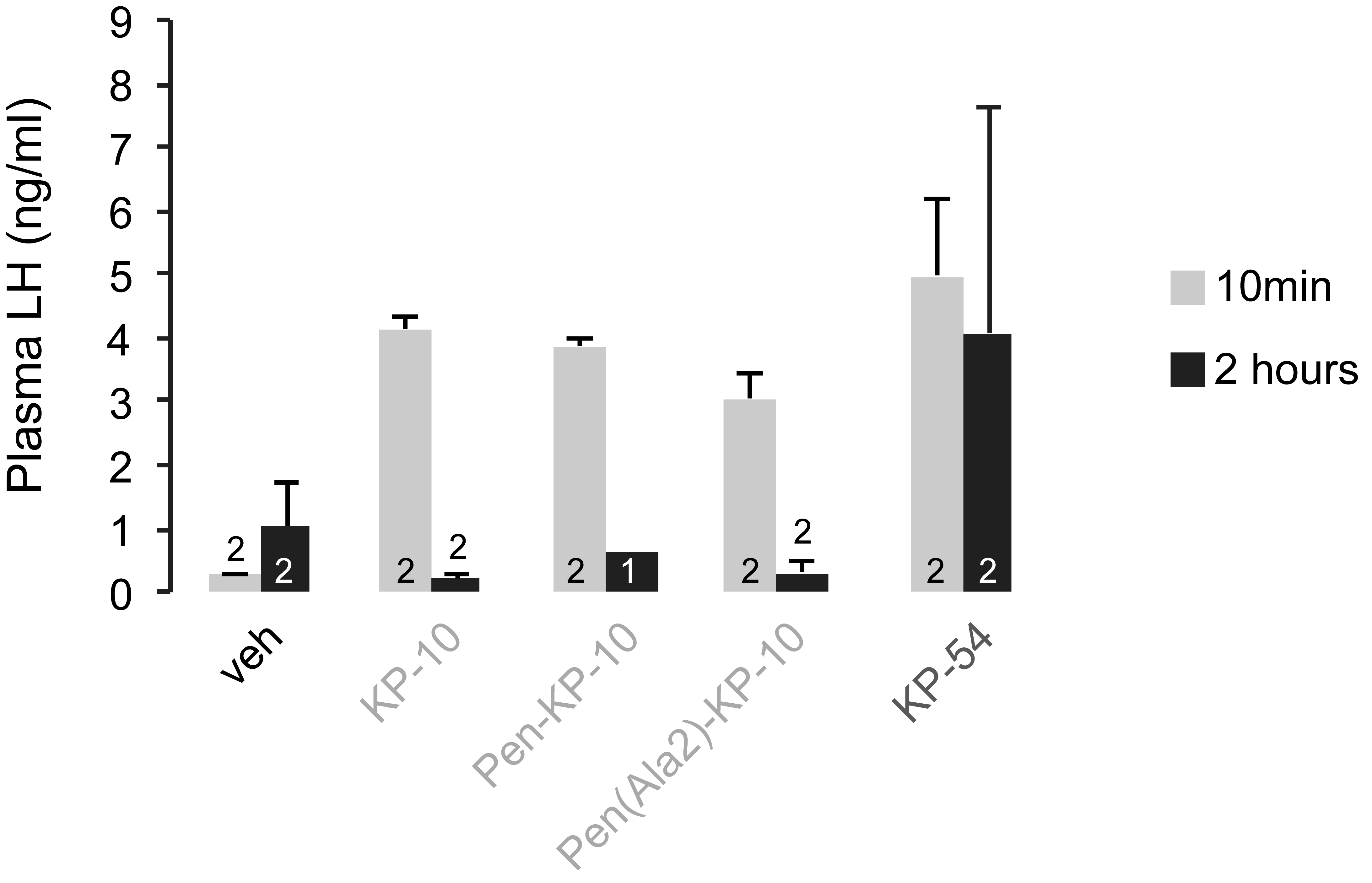

Supplement: S1 Fig — This experiment aimed at facilitating Kp10 to cross the blood-brain-barrier by adding a penetratin extension [24] to the C-terminal end of Kp10, thereby putatively provided with higher permeability. Two penetratin variants were tested: the pen(ala2) residue has a lower protein transduction domain activity than pen-Kp10. The Kp10 peptide sequence used was Y N W N S F G L R F–NH2. The peptide sequences of modified Kp10 were: N R R M K W K K Y Y N W N S F G L R F–NH2 (Pen-Kp10), and N R R M A W A K Y Y N W N S F G L R F–NH2 (Pen(ala2)-Kp10). Each animal received a single 100 μl i.p. injection of either PBS, Kp10, Kp54, Pen-Kp10 or Pen(ala2)-Kp10. Blood samples were collected at 10 min (from tail vein) and 2 hrs (from vena cava upon sacrifice) time points after administration. The results from two animals per treatment (n = 2), suggest that adding a penetratin residue to the Kp10 decapeptide does not improve the Kp10-induced LH secretion. (TIF) [file pone.0176821.s001.tif]
